# Draft Genome Sequence of Glycoside Hydrolase-Producing Trichoderma asperellum Strain IC-1

**DOI:** 10.1128/MRA.00958-20

**Published:** 2020-10-01

**Authors:** Hiro Takahashi, Erika Sakagawa, Ryosuke Toyoda, Taichiro Motomura, Masataka Murase, Anna Takahashi, Shuichi Fukuyoshi, Shin Kanamasa

**Affiliations:** a Graduate School of Medical Sciences, Kanazawa University, Kanazawa, Ishikawa, Japan; b Graduate School of Horticulture, Chiba University, Matsudo, Chiba, Japan; c Fundamental Innovative Oncology Core Center, National Cancer Center, Tokyo, Japan; d Graduate School of Bioscience and Biotechnology, Chubu University, Kasugai, Aichi, Japan; e Faculty of Information Technologies and Control, Belarusian State University of Informatics and Radio Electronics, Minsk, Belarus; f College of Bioscience and Biotechnology, Chubu University, Kasugai, Aichi, Japan; g Institute of Medical, Pharmaceutical and Health Sciences, Kanazawa University, Kanazawa, Ishikawa, Japan; Vanderbilt University

## Abstract

Glycoside hydrolases capable of degrading lignocellulose are important for effectively utilizing cellulosic biomass as a next-generation chemical resource. Trichoderma asperellum IC-1 produces various glycoside hydrolases. Here, we report a draft genome sequence of *T. asperellum* IC-1 to better understand its gene structures and gene regulatory mechanisms.

## ANNOUNCEMENT

Lignocellulose, an insoluble polysaccharide, is the most abundant renewable biomass on Earth (1). The hydrolysis of lignocellulose by glycoside hydrolases of microorganisms is the first step in utilizing lignocellulose as a resource for biofuels and biomaterials and is catalyzed by a cellulase complex containing at least three types of enzymes, endo-1,4-β-d-glucanase, exo-1,4-β-d-glucanase (cellobiohydrolase), and β-glucosidase (2). Previously, we reported that Trichoderma asperellum IC-1, a filamentous fungus isolated from soil, produces a number of extracellular cellulose- and hemicellulose-degrading enzymes; cellulose induces the production of these enzymes (3, 4). As the genes encoding these enzymes and their transcription control mechanisms remained unknown, we sequenced the genome of *T. asperellum* IC-1 in this study.

IC-1 cells were cultured in potato dextrose broth at 30°C and homogenized using a bead crusher; genomic DNA was isolated using a NucleoSpin Plant II kit (TaKaRa Bio, Inc.). The DNA library was prepared using a DNA library preparation kit (Beijing Genomics Institute, Shenzhen, China). DNA fragmentation was performed by using g-TUBE (Covaris, Inc.) to produce fragments with an average length of 300 bp, followed by end repair, A tailing, adaptor ligation, and PCR; DNA library purification was performed according to the manufacturer’s instructions. Paired-end sequencing on the DNBSEQ-G400 platform (MGI Tech, Shenzhen, China) generated 41,198,750 reads (150-bp reads). Primer sequences were removed, and low-quality reads were trimmed from the obtained short reads using fastp version 0.19.10 (5) with default parameters. *De novo* sequence assembly was performed using SPAdes version 3.14.0 (6) with the “merged” and “isolate” parameters enabled. A reference-guided scaffolding of the draft genome was conducted using RaGOO version 1.1 (7) with default parameters and the genome sequence of *T. asperellum* CBS 433.97 (GenBank accession number GCA_003025105.1). Among the RaGOO-generated scaffolds, a concatenated nonlocalized scaffold consisting of short, highly fragmented contigs without homology to the reference sequence was discarded. The resulting genome assembly was 36,071,795 bp long and was divided into 72 scaffolds comprising 201 contigs. The *N*_50_ values (contig and scaffold), GC content, and genome coverage were 628,795 bp and 2,555,590 bp, 48.3%, and 163.8×, respectively. To optimize the parameters of AUGUSTUS version 3.3.3 (8) for *T. asperellum* gene prediction, a gene model from the *T. asperellum* CBS 433.97 genome was used as reference for training the AUGUSTUS program according to the AUGUSTUS authors’ protocols (9). Among the 290 benchmarking universal single-copy ortholog (BUSCO) genes, 99.3% (including 0.0% duplicated genes) were found in the assembly, as calculated with BUSCO version 3.1.0 (10) using the trained AUGUSTUS and “fungi_odb9” data set. The coding regions for the scaffolds were predicted using the trained AUGUSTUS data set with the “noInFrameStop=true” and “genemodel=complete” parameters enabled. The estimated number of genes in the draft genome was 8,803. Gene functional annotation was performed using Trinotate version 3.2.1 (11) with default parameters.

This genomic information may provide insights into the genetic basis of gene structures and gene regulatory mechanisms in *T. asperellum* IC-1.

### Data availability.

The draft genome sequence and gene annotation for *Trichoderma asperellum* IC-1 are deposited in GenBank/ENA/DDBJ under the accession number BLZH01000000 (BLZH01000001 to BLZH01000072 for scaffolds 1 to 72). The SRA/DRA/ERA accession number is DRA010487.
